# Dysfunction of the glutamatergic photoreceptor synapse in the P301S mouse model of tauopathy

**DOI:** 10.1186/s40478-022-01489-3

**Published:** 2023-01-11

**Authors:** L. Arouche-Delaperche, S. Cadoni, C. Joffrois, G. Labernede, M. Valet, Q. César, J. Dégardin, S. Girardon, C. Gabriel, S. Krantic, S. Picaud

**Affiliations:** 1 INSERM, CNRS, Institut de La Vision, Sorbonne Université, 17 Rue Moreau, 75012 Paris, France; 2 Institut de Recherches Servier, 125 Chemin de Ronde, 78290 Croissy-Sur-Seine, France; 3 Institut de Recherches Internationales Servier, 50 Rue Carnot, 92284 Suresnes, France; 4 INSERM UMRS 938, Centre de Recherche Saint Antoine, CRSA, Sorbonne Université, 184 Rue Faubourg St Antoine, 75012 Paris, France

**Keywords:** Retina, Phosphorylated tau, Alzheimer’s disease, P301S mouse, Synapsis

## Abstract

Tauopathies, including Alzheimer’s disease, are characterized by retinal ganglion cell loss associated with amyloid and phosphorylated tau deposits. We investigated the functional impact of these histopathological alterations in the murine P301S model of tauopathy. Visual impairments were demonstrated by a decrease in visual acuity already detectable at 6 months, the onset of disease. Visual signals to the cortex and retina were delayed at 6 and 9 months, respectively. Surprisingly, the retinal output signal was delayed at the light onset and advanced at the light offset. This antagonistic effect, due to a dysfunction of the cone photoreceptor synapse, was associated with changes in the expression of the vesicular glutamate transporter and a microglial reaction. This dysfunction of retinal glutamatergic synapses suggests a novel interpretation for visual deficits in tauopathies and it highlights the potential value of the retina for the diagnostic assessment and the evaluation of therapies in Alzheimer’s disease and other tauopathies.

**Supplementary Information:**

The online version contains supplementary material available at 10.1186/s40478-022-01489-3.

## Introduction

Tauopathies include Alzheimer’s disease (AD), corticobasal degeneration (CBD), progressive parasupranuclear palsy (PSP) and frontotemporal dementia (FTD) [63]. They are characterized by an accumulation of pathological forms of tau proteins in intracellular neurofibrillary tangles (NFTs), compromising neuronal functions. The most affected neuronal functions include those supported by the cytoskeleton and axonal transport, such as synaptic transmission. It has been hypothesized that the presence of specific abnormal or hyperphosphorylated tau (p-tau) epitopes prevents the interaction of tau with microtubules, leading to the neuronal cytoskeleton collapse typical of tauopathies [57]. In addition to tauopathy, an accumulation of amyloid proteins (amyloidosis), such as amyloid-beta (Aβ), can be observed not only in AD [37], but also in CBD [5], and FTD [64].

Synaptic dysfunctions appear as very early symptoms in AD and other tauopathies [30]. Such dysfunctions may manifest as hippocampal hyperactivity in AD patients [3, 30]. The associated network abnormality has been linked to dysfunctions of inhibitory interneurons [44] in experiments on mice overexpressing the human amyloid precursor protein (APP) [43]. However, glutamatergic transmission is also affected by high Aβ levels [44]. This hyperexcitability is associated with seizures that can be treated with the antiepileptic drug levetiracetam, which improves memory task performances [3]. The abolition of tau expression in multiple animal models of AD expressing hAPP has been shown to prevent the development of epileptiform activity [53, 67]. Conversely, mice bearing tau mutations [19, 27, 52] display hippocampal neuronal hyperexcitability during the early stages of tauopathy. However, tau-related dysfunctions, like those for Aβ, affect GABAergic neurotransmission in addition to glutamatergic synapses [58]. A synergistic contribution of Tau and Aβ to neuronal hyperexcitability in AD has recently been recognized [30].

In addition to its classical neuropathological hallmarks, AD has been associated with symptoms affecting the visual system and translating into impairments of visual acuity, color vision, contrast sensitivity, visual field depth and motion perception [7]. Optical nerve and retinal thinning, reflecting neurodegeneration, have also been observed in AD patients by optical coherence tomography (OCT), and attributed to a loss of retinal ganglion cells (RGCs) [16, 31, 46]. The functional alterations measured with a pattern electroretinogram (ERG) have provided additional evidence for RGC alterations in AD patients [29, 32]. Moreover, both major histopathological hallmarks of AD (i.e. Aβ plaques and NFTs) have been detected in post-mortem retinal examinations on human patients, with Aβ plaques more prominent in the innermost part of the retina, although some plaques were also present in the outer layers containing the photoreceptors (PR)s [31]. The impact of tau protein on the retinal circuit and the higher visual centers is less clear, despite the detection of pathological forms of tau in both plexiform layers of the human post-mortem AD retina [22] and visual cortex as seen in functional exploration studies [41] in AD patients. Moreover, the presence of aggregated tau in the optic nerve lysates coming from post-mortem AD brains has also been reported [13]. In other tauopathies, literature data is sparse however few patients suggest retinal layer thinning [50] and oculomotor disturbances in PSP patients [42]. Also, p-tau were detected in the retina of post-mortem PSP patients [56].

Studies on P301S mice aiming to distinguish more clearly between the impact of tau and amyloid have revealed the occurrence of an RGC axonopathy associated with reactive microgliosis and vasculopathy [18, 65]. Xia et al. [65] recently showed that p-tau was distributed throughout the retina in a mouse models of tauopathy in line with previously reported presence of p-tau in both retina and optic nerve soluble protein extracts coming from 5-months-old P301S mice [38]. Heavily burdened p-tau was also identified in the visual cortex of rTg4510 mouse model of tauopathy [23]. However, previous studies in either mouse model [18, 23, 38, 65] or human [13, 22, 41] all assessed only either structural alterations in one or another part of the visual system or alternatively functional impairments without monitoring in parallel for structural alterations and tau burden. Here, we investigated both functional impact of mutated and overexpressing tau using P301S mouse model on the visual system assessed as a whole, i.e. including its peripheral neurosensory input (retina), visual information transmission route (optic nerve) and brain integration center of visual information (visual cortex). We performed a comprehensive functional analysis of the visual system in the P301S mouse model at the age of 6 and 9 months, a period at which synaptic loss and microglial activation in the brain provide early manifestations of tau pathology and progression of the disease [68]. Our data demonstrate a clear alteration of retinal information processing at this early stage of disease.

## Materials and methods

### Animals

Transgenic P301S mice (line PS19) bearing a gene encoding a mutant form of the human 1N4R Tau protein isoform under the control of the mouse prion protein promotor (PrP) in a mixed genetic background B6C3F1 [2, 68] were used in this study. These mice come from The Jackson Laboratory (stock no. 008169) and were purchased from Charles River Laboratories France. Heterozygous male P301S (referred to as HE-P301S) mice and their wild-type (WT) male littermates were used. These colony is homozygous for the functional *Pde6b*^+^ allele (*Pde6b*^*rd1*^ allele has been selectively removed). The experimental protocols were approved by the local animal ethics committee (Committee Charles Darwin no. 5, registration number #7442-2016110314415874v1) and performed in accordance with directive 2010/63/EU of the European Parliament.

### Immunochemistry

After euthanasia, the retina, optical nerve and brain were fixed with 4% paraformaldehyde (Cat no. 100496, Sigma-Aldrich) in 0.1 M phosphate buffer (Cat no. P4417-100TAB, Sigma-Aldrich), pH 7.2, by cardiac perfusion, and were post-fixed by incubation in the same solution for 1 h (retinas) or overnight (brains and optical nerves). Right eyes, optic nerves and brain hemispheres were cryoprotected in sucrose (10–30% in 0.1 M phosphate buffer, pH 7.2, Cat no. 84097, Sigma-Aldrich), frozen and stored at − 80 °C until use. We prepared 12 µm-thick retina and optical nerve sections with a Leica cryostat (CM3050S) and brains were cut into 50 µm-thick sagittal sections with a microtome (HM450, Microm). Left retinas and optic nerves were fixed according to the same procedure and used to prepare the whole-retina flat-mounts. Retinas were incubated in blocking solution (1% donkey serum (Cat no. 017-000-121, Jackson Immuno-Research), 2% Triton X100 (Cat no. X100-500ML, Sigma-Aldrich), 1% Tween-20 (Cat no. P7949-100ML, Sigma-Aldrich) in phosphate buffer, pH 7.2 at room temperature for 1 h and were then incubated overnight at 4 °C with the primary antibodies listed in Additional file 7: Table S1, in the same solution. All the secondary antibodies used to detect immunolabeling were goat anti-IgG conjugated to Alexa-Fluor 488, 594 or 633 (1:750, Thermo Fisher Scientific). Immunohistochemistry procedures aimed to reveal conformational or p-tau by using MC1 and PHF1 antibodies were performed at Servier facilities.

### Image acquisition and analysis

Immunolabeled tissues were imaged with a Nanozoomer slide scanner (HT-C9600 Hamamatsu) or with an Olympus FV1000 laser-scanning confocal microscope with a 20 × or 40 × objective (UPLSAPO 20XO, NA: 0.85).

Microglial cells were quantified on confocal images of the entire retina. The images were imported into Imaris sorftware (V.9, Oxford Instruments) for analysis. Counts were performed on the Iba1 channel, selecting the area of immunopositive signal and using a set threshold to eliminate false positives; colocalization with DAPI-stained nuclei was assessed systematically, as previously described [40]. The number of microglia in a selected area was then reported.

Astrocyte cells were quantified on confocal images of the entire retina. The images were imported to Fiji software (Version:2.1.0/1,53c, National Institutes of Health, USA) for analysis. The surface occupied by GFAP immunopositive cells in this channel were analyzed.

VGlut1, VIAAT and GlutSyn fluorescence intensities were quantified with Fiji software (Version:2.1.0/1,53c, National Institutes of Health, USA) in a selected square region of interest (ROI) with an area of 320 µm^2^ from the center of the retina.

RGCs and PR counts were obtained with Imaris software (V.9, Oxford Instruments) after the selection of a square region of Interest (ROI) with an area of 500 µm^2^ in the central region of the retina, close to the optic nerve. The ROI was selected on images obtained with the Nanozoomer. Counts were performed on RPMS or cone-arrestin channels.

### Assessment of visual acuity in an optomotor test

Visual acuity was assessed with an optomotor test in 15WT and 11HE mice, as previously described [15, 51], with an OptoMotry (CerebralMechanics Inc.) device. Briefly, unrestrained mice were placed on an elevated central platform, surrounded by a computer monitor generating a visual stimulus consisting of black and white vertical stripes rotating around the animal. An overhead camera was used to obtain images of the animals’ behavior from above. The optomotor response was monitored by tracking the reflexive head movements of the animal. Spatial frequency (0.03 and 0.5 cyc/deg) were measured by systematically increasing the spatial frequency of the grating at 100% contrast and rotating the drum at a speed of 2 rpm. The calculation of the visual acuity was then obtained by the OptoMotry 1.7.7 software (CerebralMechanics Inc). All mice were assessed by the same experimenter, blind to genotype.

### In vivo visual evoked potential (VEP) recordings

Mice were anesthetized by the intraperitoneal injection of a mixture of ketamine and xylazine (ketamine 1000: 80 mg/kg and xylazine: 8 mg/kg, Rompun 2%, Bayer HealthCare). VEP recording in anesthetized rather than in vigilant animals was chosen to avoid a bias of previously reported putative impact of locomotor activity on VEP [28]. We chose anesthesia option as a precaution although a more recent study found a small impact of the amount of locomotor activity on VEP which was apparently independent of tau since observed in both Tau+ and Tau− rTg4510 model of tauopathy [45]. Pupils were dilated with drops of tropicamide (Mydriaticum, 0.5%, Thea, France). A small craniotomy (approximately 1 mm × 1 mm) was performed above the primary visual cortex of the left hemisphere, centered about 3 mm laterally and 0.5 mm rostrally to the lambda point. Extracellular recordings were performed with a 16-site silicon microprobe (electrode diameter 30 µm, spacing 50 µm, A1 × 16-5 mm-50-703, NeuroNexus Technologies). Recordings were performed in random order on 5 WT and 7 HE-P301S mice at 6 months and 5WT and 5 HE-P301S mice at 9 months. Electrodes were inserted into the primary visual cortex of mice and advanced 1000 µm into the cortex with a three-axis micromanipulator (MP285, Sutter Instruments). Visual stimuli were generated with a white LED (MNWHL4, Thorlbas, Inc.) placed 10 cm away from the right eye of the animals. Both eyes were covered with Lubrithal (Dechra Veterinary Products, Denmark). The right eye was covered with a coverslip and the left eye with black fabric. The stimuli consisted of flashes of different durations (25, 50, 100, 200 ms), repeated 100 times, at four different stimulus repetition frequencies (0.2, 1, 2 and 4 Hz, respectively). Light irradiance was varied between 0.02 and 1.57 mW/cm^2^. Extracellular signals were digitized with a 16-channel amplifier (model ME16-FAI-μPA-System, MultiChannel Systems). Recordings were further analyzed with custom-developed MATLAB (MathWorks) scripts. Briefly, the crude signal was filtered between 3 and 300 Hz to assess stimulus-evoked local field potentials. The latencies and amplitudes of the typical N1, P1, N2 waves of the visual evoked potentials were determined by identifying negative and positive peaks after stimulus onset.

### In vivo retinal activity recording

Electroretinograms were performed on animals at the ages of 6- (18 WT and 15 HE-P301S) and 9 months (18 WT and 20 HE-P301S), as previously described [62]. Briefly, mice were kept in the dark overnight and were anesthetized with a mixture of ketamine 1000 (80 mg/kg, Axience, France) and xylazine (8 mg/kg, Rompun 2%, Bayer HealthCare, France). Pupils were dilated with tropicamide (Mydriaticum 0.5%, Thea, France) and phenylephrine (Neosynephrine 5%, CSP, France). The cornea was anesthetized by the local application of oxybuprocaine chlorhydrate (Thea, France). The upper and lower lids were retracted to keep the eyes open and bulging.

A small gold wire loop electrode in contact with the cornea through a layer of Lubrithal (Dechra Veterinary Products, Denmark) was used to record retinal responses to light stimuli. Needle electrodes placed on the head and back of the animals were used as reference and ground electrodes, respectively. Body temperature was maintained at ~ 37 °C with a heating pad. The light stimulus was provided by a white LED lamp in a Ganzfeld stimulator (ColorDome Lab Cradle, Diagnosys LLC). Four levels of stimulus intensity (0.003, 0.3, 3 and 10 cds/m^2^) were used for scotopic ERG recordings. The responses to each stimulus presented were averaged over five flash stimulations. Photopic cone PR responses were recorded by ERG under rod PR‐suppressing background light at 20 cd/m^2^, after a 5‐min adaptation period. A stimulus intensity of 10 cd s/m^2^ was used for the light‐adapted ERGs. Each cone PR photopic ERG response was averaged over 10 consecutive flashes. Flicker ERGs were recorded at 10 and 20 Hz. Responses were amplified and filtered (1 Hz low-cutoff and 300 Hz high-cutoff filters) with a one-channel DC-/AC-amplifier. ERGs were analyzed with Espion V6 software (Diagnosys LLC).

### Retinal recordings in vitro

Mice (9 WT and 11 HE-P310S at 6 months; 12 WT and 10 HE-P310S at 9 months of age) were anesthetized and euthanized according to institutional animal care standards and retinas were isolated as previously described [9]. Isolated retinas were flattened on a poly-L-lysine-coated cellulose membrane and gently pressed against a multi-electrode array (MEA) (MEA256 60/10iR-ITO, Multichannel systems) with the RGCs facing the electrodes. Retinas were perfused continuously with Ames’ medium with L-glutamine (A1420, Sigma-Aldrich) and oxygenated with carbogen (95% O_2_/5% CO_2_) at 34 °C at a rate of approximately 2 ml/min. Recordings were performed in random order on HE-P301S and WT mice. In some experiments, 50 μM L-2-amino-4-phosphonobutyric acid (LAP-4, a group III metabotropic glutamate receptor, Tocris Bioscience, Cat no. 0103) was bath-applied for 30 min before recordings, to block the ON-bipolar neuron pathway. Full-field white light stimuli were displayed with a digital mirror device (DMD, Vialux, resolution 1024 × 768) coupled to a X-Cite® exact ultra-stable fluorescence light source (Lumen Dynamics) and were focused on the PR plane with standard optics. Light flashes of different durations (from 15 to 200 ms) and interstimulus durations (from 65 to 5000 ms) were used. Output light irradiance was also varied between 0.1 and 0.6 mW cm^−2^. The crude extracellular activity of RGCs was digitized with a 252-channel preamplifier (MultiChannel Systems). Spikes from individual neurons were sorted using SpykingCircus software [66]. RGC responses were then analyzed with custom-developed scripts written in MATLAB (MathWorks, Natick, MA, SA). RGCs were classified according to their response to light flashes. Firing rates were used to classify the neurons as ON-, ON–OFF or OFF- bipolar cells, according to the response dominance index [1, 61]. RGCs with sustained and transient responses were identified on the basis of response duration, as described by Liu et al. [36]. The latency of each cell response to stimulus ON- or OFF-set was calculated as the time between the start of the stimulus and the maximum of the derivative of spike density function. The latency of the ON- response of ON- and ON–OFF cells was measured relative to the onset of the stimulus, whereas the latency of OFF- responses of ON–OFF and OFF- cells was determined relative to the end of the stimulus.

### Statistical analysis

Statistical analysis was carried out with Prism software (Prism 7, GraphPad software, Version 7.0e). Data are represented as the mean ± standard error of the mean (SEM). Differences between two groups were analyzed in unpaired non-parametric two-tailed Mann–Whitney tests, or unpaired parametric two-tailed Welch *t* tests for the MEA and VEP recordings. Differences were considered significant for *p* values less than or equal to 0.05: *p ≤ 0.05, **p < 0.01 and ***p < 0.001.

## Results

### Expression of abnormal and hyperphosphorylated tau proteins

**Fig. 1:**
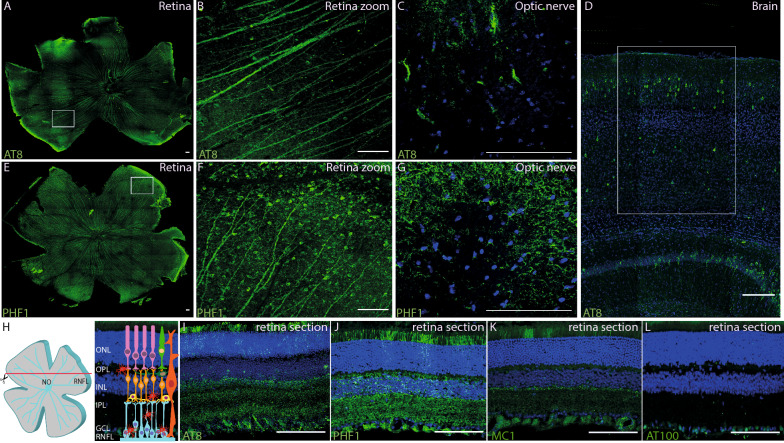
Tau-protein phosphorylation profile in the retina of 9-month-old P310S mice. **A–D, I** Immunolabeling performed with: the AT8 antibody detecting Ser202-phosphorylated tau protein; **E–G, J** the PHF1 antibody targeting the double (Ser396/Ser404)-phosphorylated tau protein; **K** the monoclonal MC1 antibody directed against the conformational epitopes formed by amino acids 7–9 and 313–322 and **L** the AT100 antibody, recognizing the double-phosphorylated residues Ser214 and Thr212. **A, B, E, F** Tau-protein labeling in whole-retina flat-mounts; **C, G** a transverse section of the optic nerve; **D** sagittal section of the visual cortex and **H–L** transverse sections of the retina. **B**, **F** Represent magnifications of the insets in **A** and **E**, respectively. All retinal flat-mounts show the labeling of the retina nerve fiber layer (RNFL) (**A, B, E, F**, in green) with the labeled fibers in **C, G** of the optic nerve (ON). (**D**, white box) The representative brain section shows neuronal labeling in the visual cortex. **I–L** Retinal sections illustrate the rather even immunolabeling of RGCs and the more variable labeling of the outer retina up to the PR level (**I–K**). The scale bars represent 100 µm

The expression of p-tau protein isoforms has been reported at 6 months in heterozygous mice, and by 1 month of age in homozygous P301S mice [18]. We studied heterozygous P301S (HE-P301S) mice. We first assessed the distribution of p-tau proteins in the retina, optic nerve and visual cortex of 9- and 6-month-old mice (Fig. 1 and Additional file 1: Fig. S1, respectively). A tau isoform phosphorylated at the Ser202 residue was detected with the AT8 antibody in the retina (Fig. 1A, B, I; Additional file 1: Fig. S1A, B), optic nerve (Fig. 1C; Additional file 2: Fig. S2) and visual cortex (Fig. 1D) of 9-month-old HE-P301S mice, but no immunolabeling was detected in WT. On flat-mounted retina, some nerve fibers formed by RGC cells running close to the surface were immunolabeled (Fig. 1A, B), and these fibers were similarly labeled within the optic nerve (Fig. 1C; Additional file 2: Fig. S2). These fibers were also AT8-immunopositive on transverse sections taken below the RGC layer (Fig. 1I). Immunolabelling was not restricted to the RGC layer. Instead, it extended to the PR inner/outer segments, inner plexiform layer (IPL) and inner nuclear layer (INL) (Fig. 1I). The PHF1 antibody, which detects p-tau at Ser396/404, provided a similar pattern of retinal and optic nerve immunolabeling (Fig. 1E-G, J), but with stronger labeling of the outer retina, and of the outer plexiform layer (OPL) in particular (Fig. 1J; Additional file 1: Fig. S1C).

Using the MC1 antibody, which specifically detects early tau protein misfolding, we also observed immunolabeling throughout the retina. More specifically, nerve fibers, the INL and a few inner/outer segments of PRs were MC1-immunopositive at both 6- (Additional file 1: Fig. S1D) and 9 (Fig. 1K) months of age. By contrast, the AT100 antibody, which recognizes the phosphorylation of the Ser214 and Thr212 residues, generated a weak immunopositive signal restricted to RGC bodies (Fig. 1L).

These data confirm previous reports of the expression of misfolded and p-tau proteins in the retina [18, 31, 38, 65], optic nerve and visual cortex, and further demonstrate that the distribution of such proteins in the retina is not restricted to the RGCs, instead extending to the inner/outer segments of PRs.

### Functional alterations in vivo

**Fig. 2:**
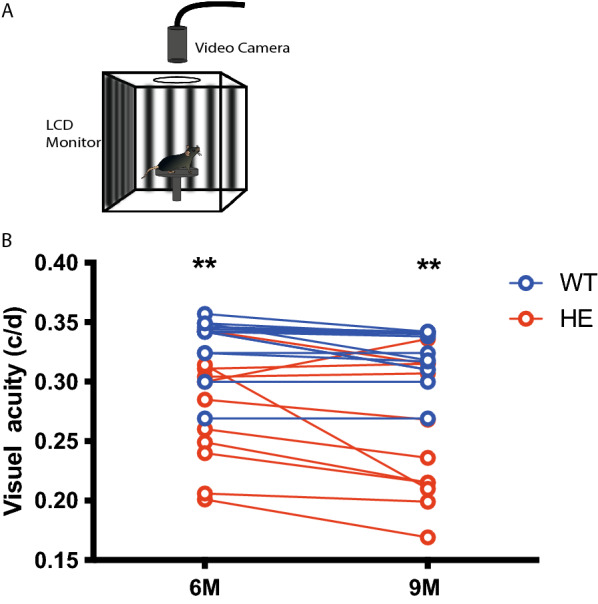
Decrease in visual acuity in HE-P301S mice. **A** Illustration of the setup used for the optomotor test. The animal is surrounded by computer screens displaying black and white stripes rotating around the animal at a given spatial frequency requiring a constant monitoring of the distance and orientation of the screens with respect to the head of the animal. The system continually adjusts stripe width for the distance of the animal’s head from the display while recording the animal’s behavior. **B** Decrease in the visual acuity of P301S mice at 6 months of age (**p = 0.002; unpaired Mann–Whitney *t* test) and nine (**p = 0.008; unpaired Mann–Whitney *t* test) (WT = 15; HE = 11mice)

We investigated whether the presence of misfolded and p-tau translated into functional impairments of the visual system, by first measuring visual acuity by evaluating the optomotor behavioral response in P301S mice. In this test, the head movements of freely moving mice following the rotation of black and white stripes on a virtual drum rotating around them at a fixed distance are assessed, as a means of evaluating visual perception. Visual acuity is determined by assessing head rotation upon the presentation of various stripe frequencies (Fig. 2A). We found that visual acuity was significantly lower in HE-P301S mice (*n* = 11) than in WT mice (*n* = 15) at both six (P301S: 0.274 ± 0.01 cyc/deg, WT: 0.32 ± 0.009 cyc/deg, *p* = 0.002) and nine (P301S: 0.25 ± 0.01 cyc/deg, WT:0.31 ± 0.01 cyc/deg, *p* = 0.008) (Fig. 2B) months of age. These results indicate an effect on the visual performances in the P301S mouse model.

**Fig. 3:**
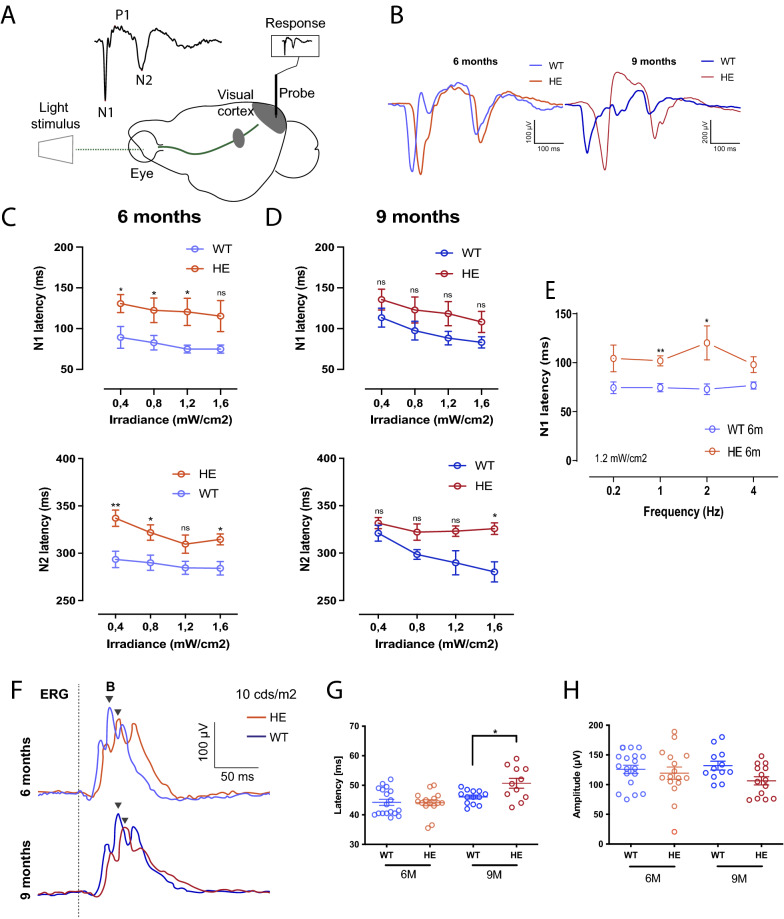
In vivo electrophysiological assessment of functional changes in the visual system in HE-P301S mice at 6 and 9 months of age. **A** Configuration for the recording of visually evoked potentials (VEP) with light stimulation of the eye and a cortical electrode. **B** Representative VEP recording showing the three typical N1, P1 and N2 waves in HE-P301S (red traces) and wild type (WT: blue traces) mice at the ages of 6 and 9 months. **C** Latencies of the N1 (top) and N2 (bottom) waves in 6-month-old and **D** 9-month-old WT- and HE-P301S mice at various light irradiance values (6 M for N1 waves: *p = 0.0418; *p = 0.0493; *p = 0.0340; for N2 waves: **p = 0.0063; *p = 0.0244; *p = 0.0116; unpaired Welch *t* test for comparison between HE-P301S and WT mice) (9 M for N1 waves: ns, *p* = 0.2370; ns, *p* = 0.2355; ns, *p* = 0.1244; for N2 waves: ns, *p* = 0.23434; ns, *p* = 0.0663; *p = 0.0147; unpaired Welch *t* test for comparison between HE-P301S and WT mice). **E** Latency of the N1 wave of 6-month-old mice at various stimulus frequencies (**p = 0.0047; *p = 0.0491; unpaired Welch *t* test). **F** Photopic ERG recordings in WT- and HE-P301S mice aged 6 and 9 months. Quantification of latencies (**G**) and amplitudes (**H**) for photopic ERG recordings for WT and HE-P301S mice at 6 and 9 months, respectively (*p* = 0.03; unpaired Welch *t* test) in photopic conditions at a light intensity of 20 cds/m^2^

We further explored visual system function by recording visual evoked potentials (VEPs) in the primary visual cortex in vivo (Fig. 3A). VEPs were recorded at the ages of 6 (7HE and 5WT animals) and 9 (5HE and 5WT animals) months, during visual stimulation of the contralateral eye. VEP amplitudes were not affected in HE-P301S mice at either age (Additional file 3: Fig. S3A), but the latencies of the typical N1 and N2 waves were delayed in HE-P301S mice relative to WT animals, at both six (Fig. 3B, C) and nine (Fig. 3B, D) months of age. In 6-month-old mice, the N1 wave of HE-P301S mice was delayed by 41.45 ± 17.36 ms relative to that of WT mice, at a light intensity of 0.4 mW/cm^2^ (*p* = 0.0418) and a similar alteration was also observed at higher light irradiance values (Fig. 3C), when the frequency of light stimulation was increased to 4 Hz (Fig. 3E). Similarly, the N2 wave was delayed by 43.54 ± 12.25 ms at 0.4 mW/cm^2^ (*p* = 0.0063) and at higher light intensities (Fig. 3D).

At 9 months of age, the differences in N1 and N2 wave latencies between WT and HE-P301S mice were not significantly different (Fig. 3D) except for the N2 latency measured at the highest light intensity (Fig. 3D). In this last case, latency was much greater in HE-P301S mice, as at 6 months of age. In addition, the differences in N1 and N2 wave latencies between 6- and 9-month-old HE-P301S mice were not statistically significant. The observed changes at 6 months, remaining the same at 9 months, indicate that this functional modification occurs at the onset of the disease (Fig. 3C, D) with possible attenuation during aging.

Then we investigated whether the VEP changes were related to retinal dysfunction or to more central changes, by recording ERGs in both photopic (light-adapted) and scotopic (dark-adapted) conditions. These measurements provided information about the functioning of retinal information processing, mostly in the outer retina. Given the well-known interindividual differences in ERG results, we performed recordings on 18 WT and 15 HE-P301S mice at the age of 6 months, and 18 WT and 20 HE-PS301P mice at the age of 9 months. In 6-month-old mice, no significant alteration to the latency or amplitude of the photopic ERG response was observed (Fig. 3F–H). By contrast, 9-month-old HE-P301S mice displayed a significant 4.3 ± 1.8 ms increase in the latency of the photopic b-wave response (HE-P301S = 50.53 ± 1.32 (*n* = 20); WT = 46.81 ± 0.77 (*n* = 18); *p* = 0.03) (Fig. 3F, G), with a non-significant decrease in peak amplitude (Fig. 3F, H). However, under scotopic conditions, no changes in amplitude or latency were observed in mice aged 6- (Additional file 3: Fig. S3B, C, D, E) or 9 (Additional file 3: Fig S3F, G, H, I) months. These ERG results suggest that in the P301S mouse model of tauopathy, functional delays in visual information processing were developing in signal transmission from cone PRs to ON-bipolar cells.

### Ex vivo retinal recordings of RGCs

We assessed the impact of abnormal p-tau on the PR-to-bipolar cell synapse further, by measuring retinal output by recording RGC spiking activity on a multielectrode array (MEA). Tau-protein deposits (AT8, PHF1, AT100 and MC1 positive cells) tended to be localized towards the central area of the retina. We therefore performed the recordings on retinal preparations from this region. Single-cell activity recordings for RGCs were isolated with a validated spike-sorting algorithm [66] for precise quantification of the impact of the disease on the various information pathways. RGC responses were classified into three different cell types on the basis of a classic response property, their response to light stimulation: ON, OFF and ON–OFF.

**Fig. 4:**
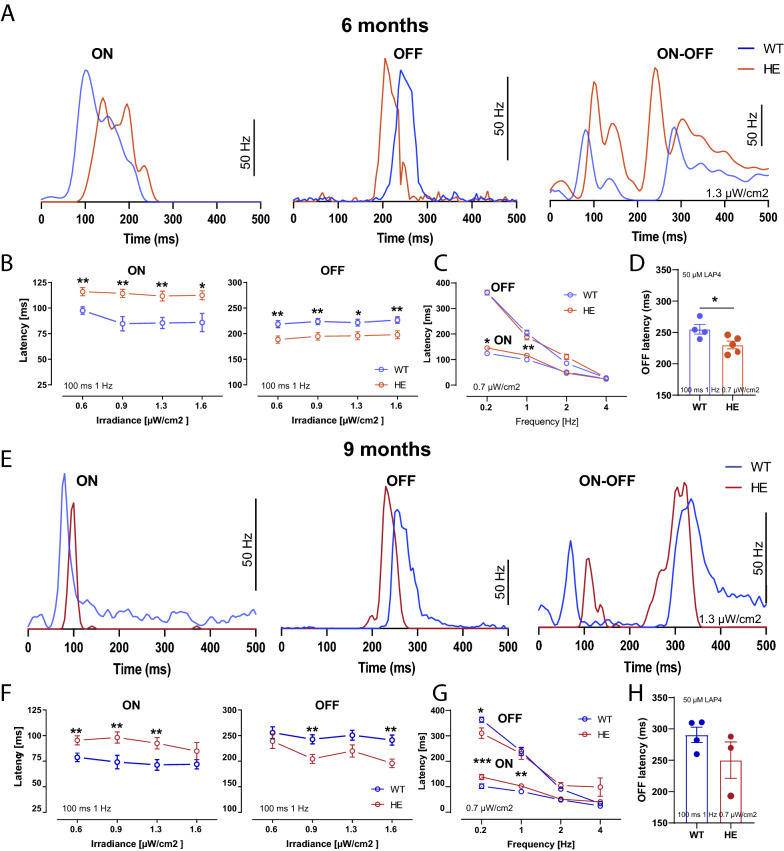
Alterations to retinal information processing in HE-P301S mice. **A** Light responses in ON, OFF and ON–OFF RGCs for WT and HE-P301S mice at 6 months of age. **B, C** Quantification of latencies for ON and OFF RGC responses in HE-P301S (*n* = 11) and WT (*n* = 9) animals at 6 months of age, for various light irradiance values (ON RGC responses: **p = 0.0043; **p = 0.0021; **p = 0.0023 *p = 0.0256; OFF RGC responses: **p = 0.00443; **p = 0.0048; *p = 0.00192; **p = 0.0082; unpaired Welch *t* test) and stimulus repetition frequencies (ON RGC cell responses: *p = 0.0299; **p = 0.0043; OFF RCG responses: *p = 0.0260; unpaired Welch *t* test). **D** OFF RGC latencies for 6-month-old WT and HE-P301 mice after the application of 50 µM LAP4 (*p = 0.423, unpaired *t* test). **E** ON, OFF and ON–OFF RGC light responses in 9-month-old HE-P301S and WT mice. **F, G** Quantification of the latencies for ON- and OFF- RGC light responses in HE-P301S (*n* = 10 retinas from *n* = 10 mice) and WT (*n* = 12 retinas from *n* = 12 mice) animals at 9 months of age, at various light irradiance values (ON RCG responses: **p = 0.0022; **p = 0.0054; **p = 0.0042; OFF bipolar cell responses: **p = 0.0065; **p = 0.0021; unpaired Welch *t* test) and stimulus repetition frequencies (ON responses: ***p = 0.0002; **p = 0.0020; OFF responses: *p = 0.0260; unpaired Welch *t* test). **H** Quantification of OFF latencies for 9-month-old WT and HE-P301S mice after the application of 50 µM LAP4

In the HE-P301S mouse retina, no change in firing frequencies relative to those of WT mice, was observed at stimulus ON-set or OFF-set (Additional file 4: Fig. S4A, B). Surprisingly, the latencies of these ON-set and OFF-set responses in HE-P301S mice were very different from those of the WT retina. Consistent with the in vivo ERG and VEP measurements, the ON responses of HE-P301S RGCs at the ON-set light stimulus (0.6 µW/cm^2^) were significantly delayed relative to WT RGCs at both 6-(P301S: 115.9 ± 4.05 ms *n* = 11 mice; WT: 97.54 ± 3.63 ms *n* = 9 mice; **p = 0.0043) and 9 (P301S: 95.36 ± 4.45 ms *n* = 10 mice; WT: 74.69 ± 4.15 ms *n* = 12 mice; **p = 0.0022) months of age (Fig. 4A, B, E, F). Similar differences were confirmed at various light irradiance values (0.9, 1.3, 1.6 µW/cm^2^).

However, the most striking difference was the significantly shorter latency of OFF-set responses in HE-P301S mice relative to WT animals at 6 months of age (6 M: HE-P301S: 188.4 ± 7.08 ms *n* = 11 mice; WT: 218 ± 6.79 ms *n* = 9 mice; **p = 0.0044, for a light intensity of 0.6 µW/cm^2^). This shorter latency of the OFF response was clearly visible for both OFF cells and ON–OFF cells, at the ages of both 6 and 9 months (Fig. 4A, B, E, F), and was statistically significant for various light irradiance values at both ages (Fig. 4B, F). However, the altered latencies were significantly different only for light stimulation at low repetition rates, at both 6 and 9 months of age (Fig. 4C, G). These results were also confirmed by a bath application of 50 µM LAP4 to block the retinal ON-bipolar neuron pathway (Fig. 4D, H).

The observed opposite effects on the ON and OFF pathways suggest that the alteration to information processing occurs at the PR terminals because, at this glutamatergic synapse, glutamate has an excitatory effect on the OFF pathway but an inhibitory effect on the ON pathway. The same alteration would therefore be expected to have opposite effects on transmission in the ON and OFF pathways, exactly as found for the studied RGC response latencies. The opposite results we obtained for the ON and OFF pathways therefore suggest that the PR synapse is affected in the retina of P301S model.

### Impaired synaptic marker expression at the retinal synapses

**Fig. 5:**
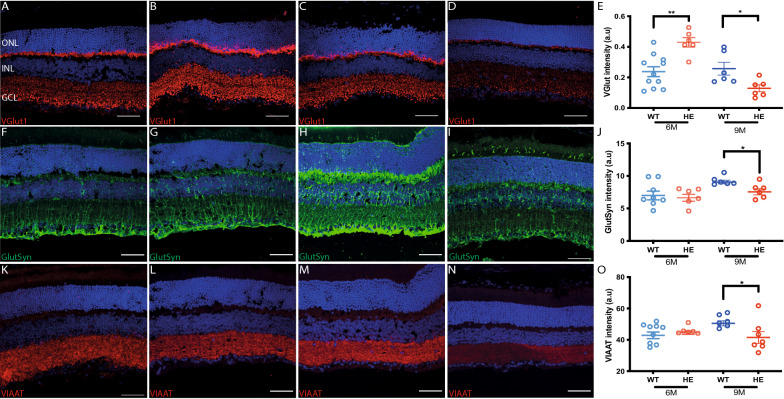
Synaptic changes in HE-P301S mice. **A–D** Distribution of VGlut1 immunolabeling in WT (**A, C**) and HE-P301S mice (**B, D**) at six (**A, B**) and nine (**C, D**) months of age, showing an increase in expression at 6 months and a decrease in expression at 9 months (**E**). **F–I** Distribution of GlutSyn immunolabeling in WT (**F, H**) and HE-P301S mice (**G, I**) at six (**F, G**) and nine (**H, I**) months of age, showing a decrease in expression at 9 months (**J**). **K–N** Distribution of VIAAT immunolabeling in WT (**K, M**) and HE-P301S (**L, N**) mice at six (**K, L**) and nine (**M, N**) months of age, showing a decrease in expression at 9 months of age (**O**). **E, J, O** Quantification of synaptic marker expression: VGlut1 (**E,** **p = 0.002, *p = 0.01), GlutSyn (**J:** *p = 0.04; Unpaired Mann–Whitney *t* test) and VIAAT (**O:** *p = 0.05; Unpaired Mann–Whitney *t *test) on retinal sections from WT and HE-P301S mice at the ages of 6 and 9 months. Fluorescence intensities were quantified in a ROI designated in the central part of the retina, close to the optic nerve. Scale bars: 50 µm

We explored the molecular mechanisms underlying PR synapse impairments, by investigating various markers of glutamatergic transmission. We first visualized the expression of vesicular transporter-1 (VGlut1) (Fig. 5A–D), which is involved in transporting glutamate into synaptic vesicles at the PR synapse [20]. Surprisingly, VGlut1 immunolabeling appeared much stronger in HE-P301S (Fig. 5B) mice than in WT animals (Fig. 5A) at the age of 6 months. This greater intensity was not limited to the outer plexiform layer (OPL), instead also extending to the inner plexiform layer (IPL) (Fig. 5A, B). The immunolabeling analysis confirmed this increase in intensity, highlighting a doubling of the intensity of VGlut1 expression throughout the retina in HE-P301S mice (Fig. 5E). However, this increase in expression was not detectable at 9 months (Fig. 5C, D), when VGlut1 expression levels in the retina of HE-P301S mice (Fig. 5D) were half those in WT mice (Fig. 5C), as shown in the quantitative analysis in Fig. 5E.

We further assessed the putative impairment of glutamate neurotransmission indicated by physiological observations, by studying the retinal expression of glutamine synthase (GlutSyn), to evaluate the involvement of the glutamate recycling pathway. In the retina, GlutSyn is expressed principally by specialized astrocytes known as Müller cells. In both WT and HE-P301S mice, the Müller cells displayed heavy immunolabeling, demonstrating their capacity to take up glutamate from the synaptic space and convert it into glutamine (Fig. 5F–I). At the age of 6 months, GlutSyn levels were similar in the two groups (WT: Fig. 5F; HE-P301S: Fig. 5G). By contrast, at 9 months of age, GlutSyn expression was significantly lower in HE-P301S (Fig. 5I) than in WT (Fig. 5H) mice (Fig. 5J), this decrease paralleling the decrease in VGlut1 expression observed in transgenic mice of the same age (Fig. 5E).

Finally, we investigated the possible involvement of the inhibitory neurotransmitters GABA and glycine in the functional impairments observed, by analyzing immunolabeling of the vesicular inhibitory GABA and glycine amino-acid transporter (VIAAT). The anti-VIAAT antibody labeled both plexiform layers in WT (Fig. 5K, M) and HE-P301S (Fig. 5L, N) animals. Quantification showed no difference between these two groups of mice at the age of 6 months (Fig. 5K versus L), whereas VIAAT expression was significantly weaker in HE-P301S (Fig. 5N) than in WT (Fig. 5M) mice at the age of 9 months (Fig. 5O).

These results indicate that a change in glutamatergic transmission at the PR synapse had occurred by the age of 6 months, and that the direction of this change had reversed at 9 months, contrasting with the functional changes. However, these results confirm the early changes in PR functional state at the PR synapse and their clear pattern of change over time, for both excitatory and inhibitory synaptic transmission in P301S model of tauopathy.

### Cell death and microgliosis

**Fig. 6:**
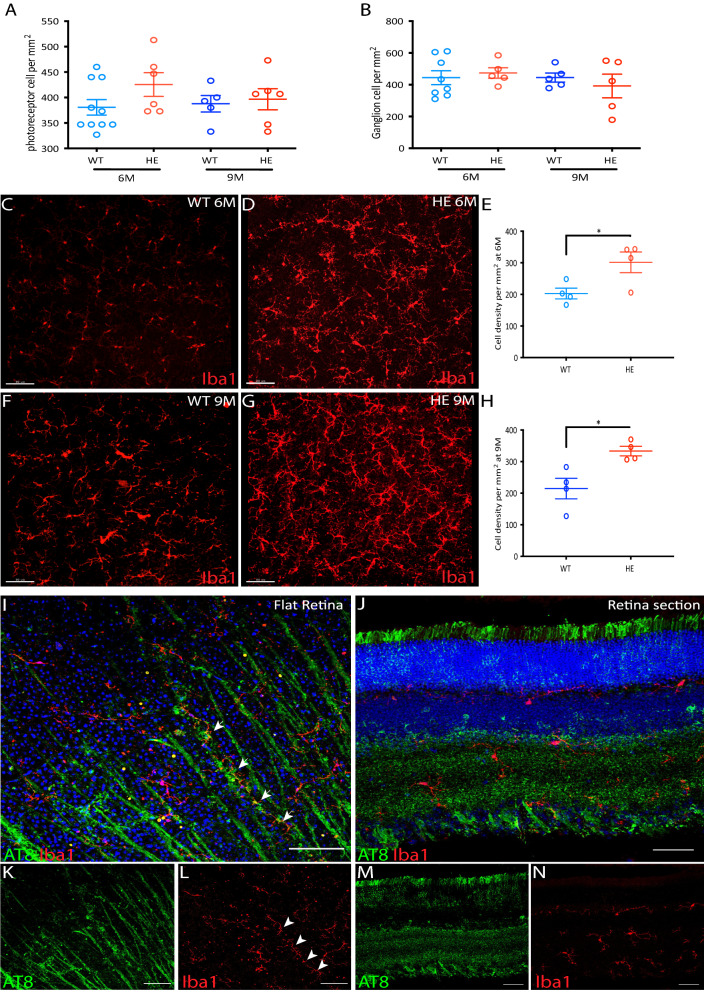
Microgliosis in the retina of P310S mice. **A, B** Quantification of cone PRs and RGCs in WT and HE-P310S mice, at 6 and 9 months of age, following immune labeling with the cone PR arrest in antibody and the RPBMS antibody, respectively. **C–H** Distribution and quantification of retinal microglia immuno labeled with the Iba-1 antibody (red) in whole-mount retinas from WT (**C, F**) and HE-P310S (**D, G**) mice aged six (**C, D**) and nine (**F, G**) months. **E, H** Quantification of retinal microglia in WT and HE-P310S mice aged six (**E**: *p = 0.05; unpaired Mann–Whitney *t* test) and nine (**H**: *p = 0.02; unpaired Mann–Whitney *t* test) months. **I–N** Microglial cell localization (red) with respect to the distribution of p-tau protein, as determined with the AT8 antibody (green) on a retinal flat-mount (**I, K, L**) and a retinal section (**J, M, N**). White arrowheads indicate microglia aligned with AT8-immunopositive RGC axons (**I, L**). Scale bars: 80 µm in **A–G**, 50 µm in **I–N**

RGC degeneration has been described in the retina and reported to be associated with a microglial reaction [21]. In view of the PR dysfunction, we investigated whether cone PRs were also affected by this process of degeneration. We therefore quantified cone PRs in HE-P301S mice at the ages of both 6 and 9 months. RGCs were quantified in parallel, this quantification revealed no significant difference (Fig. 6A, B) between HE-P301S and WT mice. The absence of neuronal degeneration was confirmed by the absence of apoptotic cell nuclei in the retina (data not shown), and by the absence of retina thinning observed during in vivo retinal imaging by OCT (Additional file 5: Fig. S5).

Microgliosis is a very sensitive marker of neuronal dysfunction that may occur before neurodegeneration. We therefore quantified the distribution of microglial cells, which have been reported to increase in number in the retina [65] and brain of P301S mice [68]. The number of microglial cells in retinas flat-mounts from HE-P301S mice (301 ± 32.58 and 333.4 ± 15.16 at 6 and 9 months of age, respectively) was greater than that for WT mice (6 months: 202.7 ± 17.03, *p = 0.05; 9 months: 214.8 ± 32.49, *p = 0.02) (Fig. 6C–H). The observed microgliosis was accompanied by morphological alterations, including an enlargement of the microglia cell bodies seen close to the RGC axons in the RGC layer on retinal flat-mounts (Fig. 6I, K, L). These morphologically altered microglial cells extended up to the OPL (Fig. 6J, M, N). We further investigated whether microglia cells invaded the ONL and inner/outer PR segments, as reported in all classical models of PR neurodegeneration [62]. However, we observed no migrating microglia in the PR layers, confirming an absence of PR degeneration despite the PR dysfunction detected in ERG experiments.

We also investigated the astrogliosis in flat-mount retinas. No difference was observed in the surface coverage by GFAP-immunopositive astrocytes between HE-P301S and WT mice at either studied age (Additional file 6: Fig. S6).

## Discussion

This study provides a detailed and integral analysis of the expression of abnormal and p-tau proteins in all components of the visual system (retina, optic nerve and visual cortex) in a well characterized P301S mouse model of tauopathy [68]. We show that the distribution of pathological tau proteins in the retina is not restricted to the retinal ganglion cells, with these proteins being found right up to the inner/outer segments of photoreceptors. We also show, for the first time, that the retina is associated with visual dysfunctions stemming from changes to visual signal processing in both the retina and visual cortex. Furthermore, our study also identifies the impairments of glutamatergic neurotransmission and microgliosis already detectable at the early stage in the studied animal model of tauopathy as putative mechanisms underlying the observed visual dysfunctions. Of utmost importance, our data indicate that observed functional impairments precede structural alterations and later neurodegenerative processes.

### Alterations to the visual system

The observed functional changes to the visual system of P301S mice were detected when the animals were 6 months old, the age at which synapse loss and microglial activation are observed in the brain and are detected as early manifestations of tau pathology and disease progression in this animal model [68]. We observed alterations to synaptic transmission at the cone-PR synapse reminiscent of the development of brain neuronal excitability. These functional results are consistent with histological examinations showing the presence of pathologically p-tau proteins throughout the retina and in various areas of the brain at this early stage of disease. Our results confirm the presence of p-tau proteins in the various cell layers of the retina, the optic nerve and the visual cortex at this age. The pathological changes detected in this study included microgliosis, suggesting major neuroinflammatory changes in the retina. Microglial cells were detected close to the RGCs expressing p-tau proteins. This situation is similar to that in the brainstem and spinal cord of P301S mice, in which microglial cells surround tau-positive neurons [4]. These functional and histological changes within the retina indicate that the disease develops in a similar manner in the retina and in the various areas of the brain. The retina may, therefore, constitute an excellent tissue model for use in drug development for tauopathy and AD, due to its easy and non-invasive access for repeated imaging and recording with no vital implications for the well-being of animals and patients in long-term follow up.

Our in vivo and ex vivo electrophysiological recordings of the retina and the visual cortex provide a detailed functional analysis of early visual deficits in the HE-P301S mouse model of tauopathy. Moreover, the behavioral tests performed for the evaluation of acuity provide the first evidence that these early functional alterations translate into visual impairments that can be detected in a non-invasive manner right from the onset of tau-related disease. However, given that the pathological tau protein was found in all structures of the visual system, from the retina to the visual cortex, it is not possible to attribute the decrease in visual acuity to the retinal dysfunction. The delayed RGC response at light ON-set may contribute to the delayed VEP response, but changes to the optic nerve or visual cortex may also contribute to the delayed VEP response and behavioral changes. Nevertheless, these changes provide easily measurable parameters for longitudinal studies in living animals for drug testing purposes.

### Visual changes in tauopathies and AD

In AD patients, a significant decrease in the thickness of the retinal nerve fiber layer has been detected in the superior quadrant [6, 32], and Aβ amyloid deposits have been detected in this area of the retina [31]. A decrease in the thickness of the retina nerve fiber (RNFL) layer in the superior quadrant has been found in patients with mild cognitive impairment, but decreases in the inferior quadrant have been observed only in patients with severe AD [35]. Finally, a decrease in RNFL thickness was also reported for all quadrants (superior, inferior, nasal and temporal) in one study [46]. Functional studies have reported significant abnormalities on pattern ERG, with an increase in the latency of the P50 wave and smaller amplitudes of the P50 and N95 waves, whereas the pattern-VEP showed an increase in latency for both the P100-wave and N135 [29]. These findings were correlated with the decrease in RNFL thickness [39]. Other studies have reported only a longer latency of the P100 and N135 waves, with no statistically significant difference in the amplitudes of the P100 and N135 waves between AD patients and controls [32, 47].

Animal models of amyloidosis are the most widely used in the domain of AD. For instance, in aged APP/PS1 mice, the a- and b-wave scotopic responses were found to be altered [14, 48]. Similar abnormalities of the visual system were observed in 5xFAD models [10]. Interestingly, in Tg2575 model of amyloidosis which displays some pathological tau-related alterations but not NFT [26], Latina and colleagues demonstrated, via an elegant immunotherapy approach by using a neutralizing tau antibody against its toxic N-terminal fragment, that decreasing tau content yields abolishment of both Aβ and p-tau accumulation, as well as tuning down of some neuroinflammatory (i.e. increased GFAP but not Iba-1 expression) and synaptic (e.g. synaptophysin but not SNAP-25) impairments at the early stage of pathogenesis in the retina [33]. In models featuring AD-associated tauopathy specifically [53], such as rTg4510 transgenic mice engineered to express (Tau+) or not (Tau−) this protein, visual cortical plasticity and VEP were disturbed [45]. However, this previous study assessed only visual cortex but not retina [45] so that the origin of visual impairment (neurosensory component or central processing of the visual information) remained unknown. In another model of tauopathy, homozygous P301S mice, ERG revealed a decrease in P1, N1 peak amplitude and an increase in peak latencies [38]. Our findings for P301S mice indicate a similar decrease on pattern ERG and an increase in latency, both of which were previously attributed to RGC dysfunction [38]. Our observation of PR synapse dysfunction, with a delayed ON response at the RGC level, provides a potential alternative interpretation for the longer latencies obtained in clinical analyses of ERG and VEPs of AD patients [32, 47]. Furthermore, alterations to the regulation of glutamate concentration at the PR synapse can enhance the reported spillover at this synapse [59], thereby decreasing visual acuity, as observed here in mice. The absence of PR and RGC degeneration in P301S mice reported here is consistent with the observed absence of change in the retinal layering of the PR layers. This absence of structural alterations is consistent with our investigation of an early stage of disease, preceding RGC degeneration. Our functional investigation indicated that the early electrophysiological alterations had already led to a loss of visual acuity by the onset of tau-related disease. These results suggest that similar changes to the PR synapse in patients with AD or other tauopathies might also lead to a loss of visual acuity before RGC loss. Further studies are required to determine whether cone PR synapse dysfunction occurs in a similar manner in amyloid models of AD, in AD patients or in patients with other tauopathies.

### Synaptic changes in tauopathies

Synaptic dysfunctions including synaptic loss appear as very early symptoms in both AD and tauopathies such CBD and PSP. In particular, decreases on the SV2A presynaptic marker have been observed in such patients in vivo [24, 30]. The hippocampal hyperactivity observed in AD patients is a well-known example [3, 30]. Such hyperexcitability has also been reported at early stages of brain tauopathy in mice bearing the P301S [19, 52] or other (P301L [27] G272V [19]) tau mutations. We found that the glutamatergic synapse at the PR terminal level was affected in the P301S mouse model. The observed effect is unlikely to be postsynaptic because the ON and OFF responses are mediated by different glutamatergic receptors. Indeed, the cone PR terminal tonically releases glutamate in the dark, this release being stopped by light exposure. Consequently, the ON response at this synapse is mediated by the mopping up of glutamate from the synaptic cleft. The longer latency of the ON responses reflects a higher glutamate concentration in the PR synaptic cleft in the dark due to higher levels of release, or lower levels of uptake. Conversely, at the light OFF-set, glutamate release from vesicles resumes, depolarizing OFF bipolar cells to generate the OFF-response. The shorter latency of the retinal OFF response requires a faster diffusion of the released glutamate to flat contact of OFF bipolar cells. This is also consistent with higher levels of glutamate release from vesicles, or lower levels of glutamate uptake. At the cone PR synapse, the glutamate transporter may control not only glutamate release [49], but also glutamate diffusion to the flat contacts of OFF bipolar cells [17, 54]. Higher concentrations of glutamate in the synaptic cleft have also been reported at the hippocampal synapse in a mouse model carrying the A152T-variant of human tau (htau-A152T) [11]. These data indicate that the p-tau could induce a change in the kinetics of glutamate flux at the glutamatergic synapse, with glutamate concentration in the synaptic cleft increasing more rapidly at light OFF-set and decreasing more slowly at light ON-set.

The increase in VGlut1 levels in PR terminals at the age of 6 months is consistent with higher levels of glutamate release, leading to faster replenishment at the synapse and, thus, a shorter OFF latency and in line with recently published data using another mouse model of AD-like pathology (Tg2576) to assess some features of tauopathy [33]. By contrast, the higher glutamate concentration in the synaptic cleft at light ON-set would require a longer washout time. This hypothesis is supported by the observed increase in stimulation frequency in the P301S mouse line studied at the age of 6 months, abolishing the differences in latency in the ON- and OFF- pathways, probably through exhaustion of the pool of docked synaptic vesicles. However, this enhanced transmitter release in the context of pathological tau protein expression conflicts with the reported inhibition of vesicular release by tau binding to the vesicles [69]. Furthermore, the lower levels of VGlut1 expression at 9 months despite a similar increase in the latency of the ON response and a shorter latency of the OFF response, suggests that the tauopathy affects glutamate transporters in either Müller glial cells or cone PRs. A similar inhibition of glutamate transport was previously described for Aβ peptides [34] and for p-tau [25]. Interestingly, at least in the hippocampus of the TgCRND8 mouse model of AD-related amyloidosis, this early hyperexcitability is followed by hypoexcitability and impaired neuronal function by the time the disease reaches the full-blown stage [8]. These findings are reminiscent of our findings for VGlut1 in the retina. Our data are consistent with the notion that different tau mutations systematically perturb glutamate neurotransmission at the presynaptic level, leading to either hyper- or hypoexcitability [12]. Further studies are required to investigate the molecular changes occurring at the cone PR synapses as a consequence of the tauopathy.

The development of new therapeutic approaches for AD and other tauopathies is currently limited by difficulties identifying early stages of the disease and assessing the physiological effects of new drugs on disease progression. Recently, new diagnostic approaches assessing blood biomarkers have emerged. However, they do not reflect the neuronal functional state [60]. Here, we propose to take advantage of the retina to generate an early functional diagnosis strategy and for the non-invasive response follow up to therapeutic treatments. The retina is a relatively easily accessible part of the central nervous system for both imaging and cell recording. Various tools are available for assessing structural alterations to the retina [55] and the physiology of visual information processing by the retina (ERG, VEP recordings) in both animal models and in humans. Amyloid burden and tau aggregation share similar effects on the visual system in animal models and humans [29, 32, 38, 39]. The retina could, therefore, be used in a non-invasive translational strategy not only for drug development in animal models, assessing functional evaluations as proposed above, but also for early disease pre-screening of subjects at risk, diagnosis and monitor longitudinally treatment efficacy in patients with AD or other tauopathies. It could be interesting to evaluate longitudinally such retinal parameters in well characterized subjects at risk and in patients with tauopathies to evaluate their potential interest for future clinical trials.

## Supplementary Information

**Additional file 1: Fig. S1**. Distribution of phosphorylated Tau in the retina. (**A**) Flat-mount retina from a six-month-old HE-P301S mouse, immunolabeled with AT8. (**1A**) Magnification of region 1 in (**A**), which is nearest to the optic nerve, followed by 2A and then 3A. (**1B**). Magnification of region 1, which is nearest to the optic nerve, followed by 2B and 3B of region (**B**), which shows immunolabeling with PHF1 (**C**) or MC1 (**D**) antibodies for all ages. (**E**) Magnification of a flat-mount retina from a nine-month-old WT mouse, immunolabeled with AT8. Scale bar=100 um.

**Additional file 2: Fig. S2**. Expression of phosphorylated Tau in the optical nerve of P301S mice at the age of nine months. Transverse section of the optical nerve immunolabeled with: AT8 (**A**); AT100 (**B**); MC1 (**C**) and PHF1 (**D**). Scale bar=50 um.

**Additional file 3: Fig. S3**. Amplitude of the VEP and ERG responses under scotopic conditions in P301S mice. (**A**) N1 negative peak amplitude of VEP recordings at increasing light irradiance values for six- and nine-month-old WT and HE-P301S mice. (**B**) Scatter plot of a-waves recorded at different light intensities (0.003, 0.03, 0.3, 3 and 10cd.s/m2) in six-month-old mice. (**C**) Differences in scotopic a-wave with different light intensities, the mean of each group of mice is shown (WT: blue; HE-P301S: red). (**D**) Scatter plot of b waves. (**E**) Mean b-waves from six-month-old WT- and HE-P301S mice for each light intensity. (**F**) Measurements of a-waves in nine-month-old mice, at different light intensities (**G**) Mean a-wave measurements in nine-month-old mice. (**H**) Scatter plot of b-waves measured in nine-month-old mice at different light intensities. (**I**) b-wave means for each group of WT- and HE-P301S mice, for the different light intensities.

**Additional file 4: Fig. S4**. Retinal MEA recordings. (**A**) Mean firing rate for the ON and (**B**) OFF responses of RGCs at various light irradiance values, for retinas from six- and nine-month-old WT and HE-P301S mice.

**Additional file 5: Fig. S5**. In vivo optical coherence tomography (OCT) imaging in P301S mice. (**A**) Micrographs for nine-month-old WT and HE-P301S mice, showing the different layers of the retina. (**B**) Layer thicknesses (GCL+IPL; GCL+OPL; GCL+ONL; and the whole retina) were analyzed with Image-J software. No significant differences were observed between the different layers.RNFL: retina nerve fiber layer, GCL: ganglion cell layer, IPL: inner plexiform layer, INL: inner nuclear layer, OPL: outer plexiform layer, ONL: outer nuclear layer.

**Additional file 6: Fig. S6**. Absence of astrogliosis in the retina of P301S mice. (**A**) Surface coverage by GFAP-immunopositive astrocytes in flat-mount retinas from WT (**A**,** D**) and HE-P301S (**B**,** E**) mice at six- (**A**,** B**) and nine- (**D**,** E**) months of age. Quantification of the surface occupied by GFAP-immunopositive cells at six (**C**) and nine (**F**) months of age. Scale bar=50μm.

**Additional file 7: Table S1**. List of antibodies used in the immunofluorescence study.

## Data Availability

The data that support the findings of this study are available from the corresponding author upon reasonable request.

## Abbreviations

AD: Alzheimer’s disease
Aβ: Amyloid-beta
APP: Amyloid precursor protein
CBD: Corticobasal degeneration
ERG: Electroretinogram
FTD: Frontotemporal dementia
GlutSyn: Glutamine synthase
INL: Inner nuclear layer
IPL: Inner plexiform layer
MEA: Multielectrode array
NFTs: Neurofibrillary tangles
OCT: Optical coherence tomography
ON: Optic nerve
p-tau: Phosphorylated tau
PR: Photoreceptor
PSP: Progressive parasupranuclear palsy
RGC: Retinal ganglion cell
RNFL: Retina nerve fiber layer
RPBMS: RNA-binding protein with multiple splicing
VGlut1: Vesicular transporter 1
VIAAT: Vesicular inhibitory GABA and glycine amino-acid transporter
