# Supplementary material for: Dysfunction of the glutamatergic photoreceptor synapse in the P301S mouse model of tauopathy
Source: Acta Neuropathol Commun. 2023 Jan 11;11:5. doi: 10.1186/s40478-022-01489-3 (PMC9832799; doi:10.1186/s40478-022-01489-3)
Supplement: Supplementary file 2 — Additional file 2: Fig. S2. Expression of phosphorylated Tau in the optical nerve of P301S mice at the age of nine months. Transverse section of the optical nerve immunolabeled with: AT8 (A); AT100 (B); MC1 (C) and PHF1 (D). Scale bar=50 um. [file 40478_2022_1489_MOESM2_ESM.pdf]

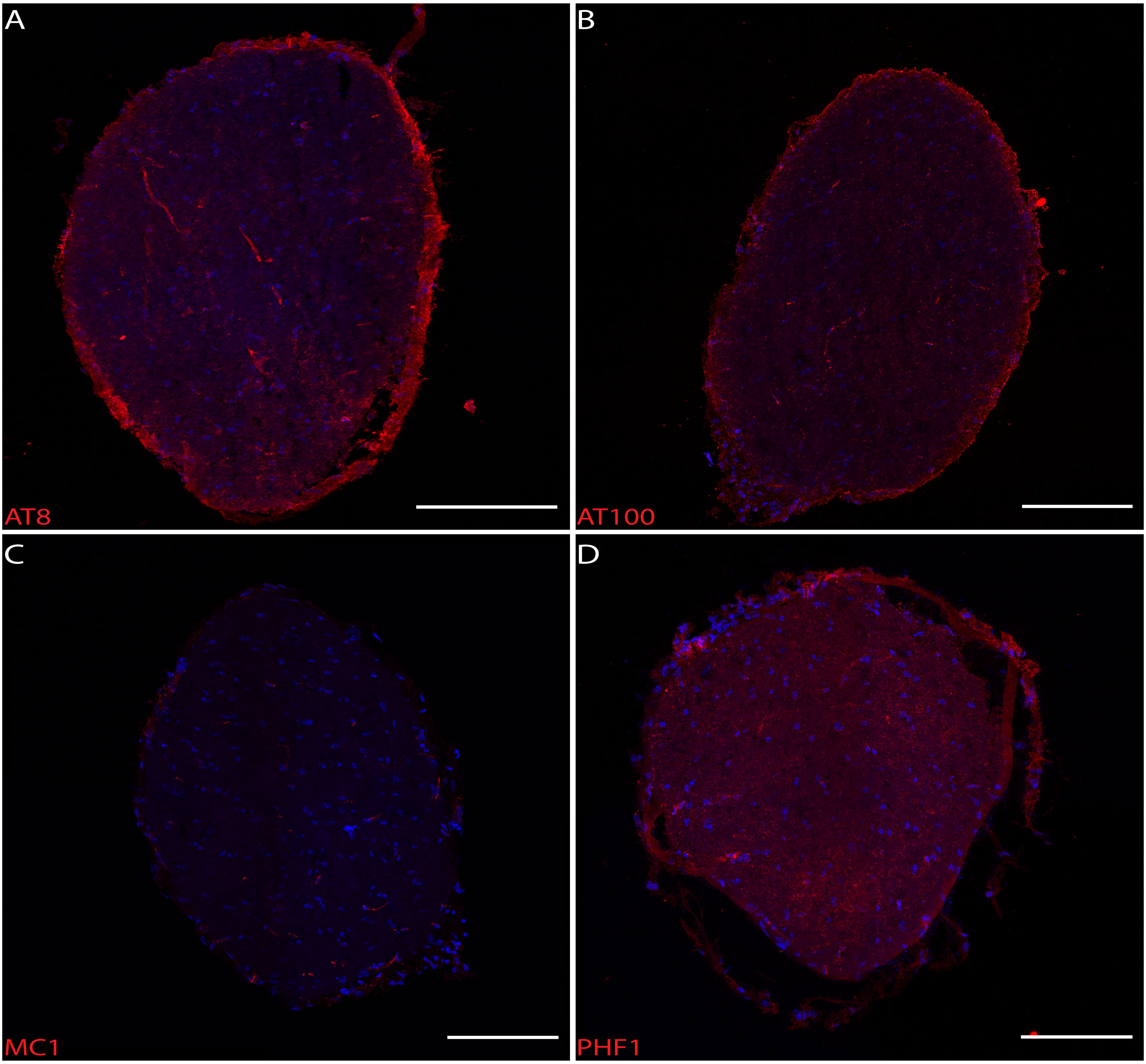

Additional file 2: Fig. S2. Expression of phosphorylated Tau in the optical nerve of P301S mice at the age of nine months. Transverse section of the optical nerve immunolabeled with: AT8 (A); AT100 (B); MC1 (C) and PHF1 (D). Scale bar=50 um.
