# Supplementary material for: Dysfunction of the glutamatergic photoreceptor synapse in the P301S mouse model of tauopathy
Source: Acta Neuropathol Commun. 2023 Jan 11;11:5. doi: 10.1186/s40478-022-01489-3 (PMC9832799; doi:10.1186/s40478-022-01489-3)
Supplement: Supplementary file 3 — Additional file 3: Fig. S3. Amplitude of the VEP and ERG responses under scotopic conditions in P301S mice. (A) N1 negative peak amplitude of VEP recordings at increasing light irradiance values for six- and nine-month-old WT and HE-P301S mice. (B) Scatter plot of a-waves recorded at different light intensities (0.003, 0.03, 0.3, 3 and 10cd.s/m2) in six-month-old mice. (C) Differences in scotopic a-wave with different light intensities, the mean of each group of mice is shown (WT: blue; HE-P301S: red). (D) Scatter plot of b waves. (E) Mean b-waves from six-month-old WT- and HE-P301S mice for each light intensity. (F) Measurements of a-waves in nine-month-old mice, at different light intensities (G) Mean a-wave measurements in nine-month-old mice. (H) Scatter plot of b-waves measured in nine-month-old mice at different light intensities. (I) b-wave means for each group of WT- and HE-P301S mice, for the different light intensities. [file 40478_2022_1489_MOESM3_ESM.pdf]

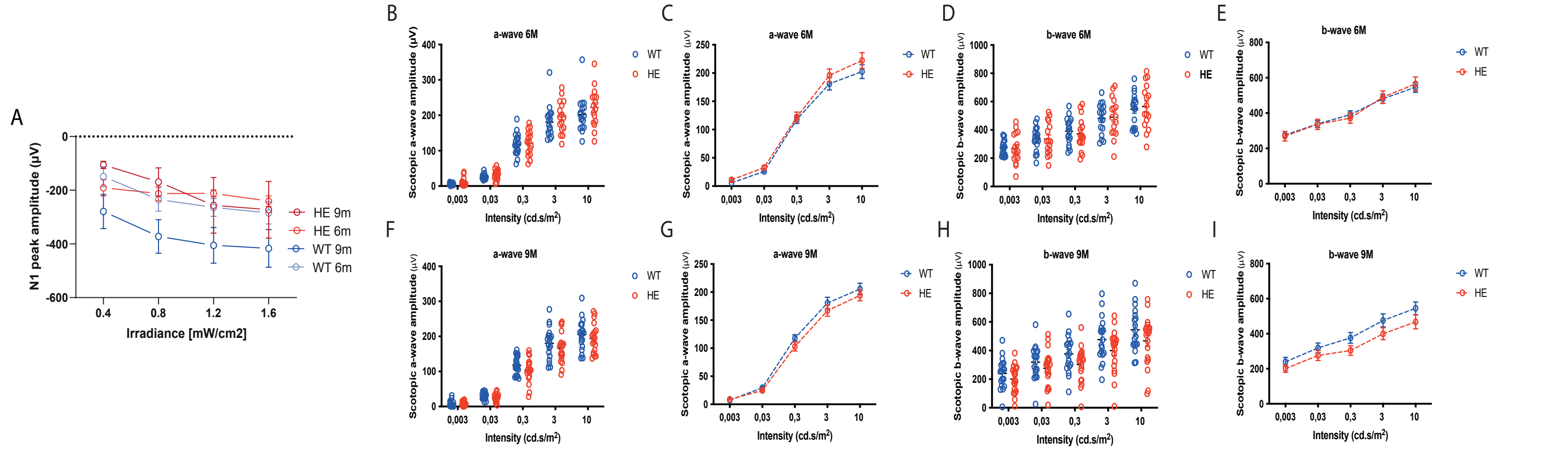

Additional file 3: Fig. S3. Amplitude of the VEP and ERG responses under scotopic conditions in P301S mice. (A): N1 negative peak amplitude of VEP recordings at increasing light irradiance values for six- and nine-month-old WT and HE-P301S mice. (B): Scatter plot of a-waves recorded at different light intensities (0.003, 0.03, 0.3, 3 and 10cd.s/m2) in six-month-old mice. (C) Differences in scotopic a-wave with different light intensities, the mean of each group of mice is shown (WT: blue; HE-P301S: red). (D): Scatter plot of b waves. (E) Mean b-waves from six-month-old WT- and HE-P301S mice for each light intensity. (F): Measurements of a-waves in nine-month-old mice, at different light intensities (G) Mean a-wave measurements in nine-month-old mice. (H): Scatter plot of b-waves measured in nine-month-old mice at different light intensities. (I) b-wave means for each group of WT- and HE-P301S mice, for the different light intensities.
