# Supplementary material for: Dysfunction of the glutamatergic photoreceptor synapse in the P301S mouse model of tauopathy
Source: Acta Neuropathol Commun. 2023 Jan 11;11:5. doi: 10.1186/s40478-022-01489-3 (PMC9832799; doi:10.1186/s40478-022-01489-3)
Supplement: Supplementary file 4 — Additional file 4: Fig. S4. Retinal MEA recordings. (A) Mean firing rate for the ON and (B) OFF responses of RGCs at various light irradiance values, for retinas from six- and nine-month-old WT and HE-P301S mice. [file 40478_2022_1489_MOESM4_ESM.pdf]

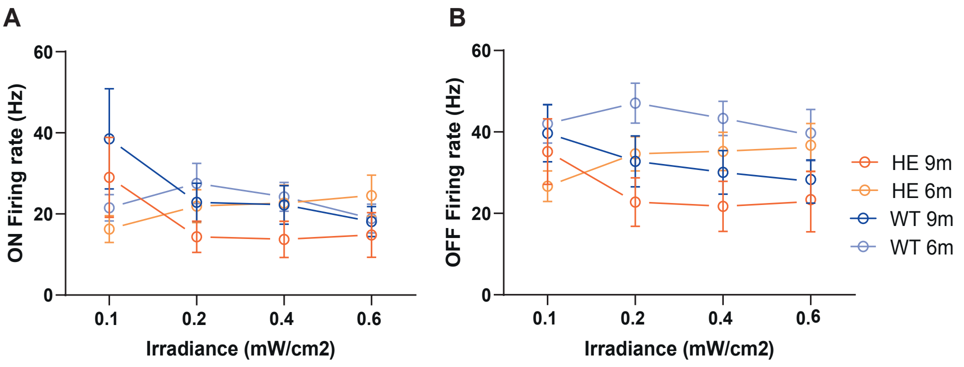

Additional file 4: Fig. S4. Retinal MEA recordings. (A): Mean firing rate for the ON and (B): OFF responses of RGCs at various light irradiance values, for retinas from six- and nine-month-old WT and HE-P301S mice.
