# Supplementary material for: Dysfunction of the glutamatergic photoreceptor synapse in the P301S mouse model of tauopathy
Source: Acta Neuropathol Commun. 2023 Jan 11;11:5. doi: 10.1186/s40478-022-01489-3 (PMC9832799; doi:10.1186/s40478-022-01489-3)
Supplement: Supplementary file 5 — Additional file 5: Fig. S5. In vivo optical coherence tomography (OCT) imaging in P301S mice. (A) Micrographs for nine-month-old WT and HE-P301S mice, showing the different layers of the retina. (B) Layer thicknesses (GCL+IPL; GCL+OPL; GCL+ONL; and the whole retina) were analyzed with Image-J software. No significant differences were observed between the different layers.RNFL: retina nerve fiber layer, GCL: ganglion cell layer, IPL: inner plexiform layer, INL: inner nuclear layer, OPL: outer plexiform layer, ONL: outer nuclear layer. [file 40478_2022_1489_MOESM5_ESM.pdf]

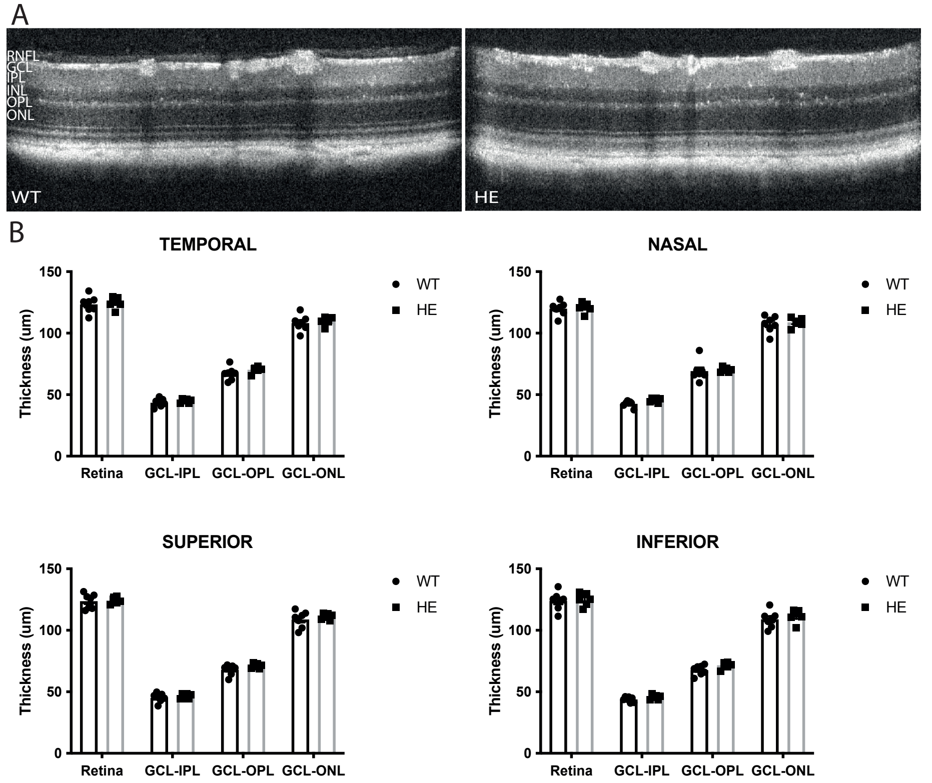

Additional file 5: Fig. S5. In vivo optical coherence tomography (OCT) imaging in P301S mice. (A): Micrographs for nine-month-old WT and HE-P301S mice, showing the different layers of the retina. (B): Layer thicknesses (GCL+IPL; GCL+OPL; GCL+ONL; and the whole retina) were analyzed with Image-J software. No significant differences were observed between the different layers. RNFL: retina nerve fiber layer, GCL: ganglion cell layer, IPL: inner plexiform layer, INL: inner nuclear layer, OPL: outer plexiform layer, ONL: outer nuclear layer.
