# Supplementary material for: Dysfunction of the glutamatergic photoreceptor synapse in the P301S mouse model of tauopathy
Source: Acta Neuropathol Commun. 2023 Jan 11;11:5. doi: 10.1186/s40478-022-01489-3 (PMC9832799; doi:10.1186/s40478-022-01489-3)
Supplement: Supplementary file 6 — Additional file 6: Fig. S6. Absence of astrogliosis in the retina of P301S mice. (A) Surface coverage by GFAP-immunopositive astrocytes in flat-mount retinas from WT (A, D) and HE-P301S (B, E) mice at six- (A, B) and nine- (D, E) months of age. Quantification of the surface occupied by GFAP-immunopositive cells at six (C) and nine (F) months of age. Scale bar=50μm. [file 40478_2022_1489_MOESM6_ESM.pdf]

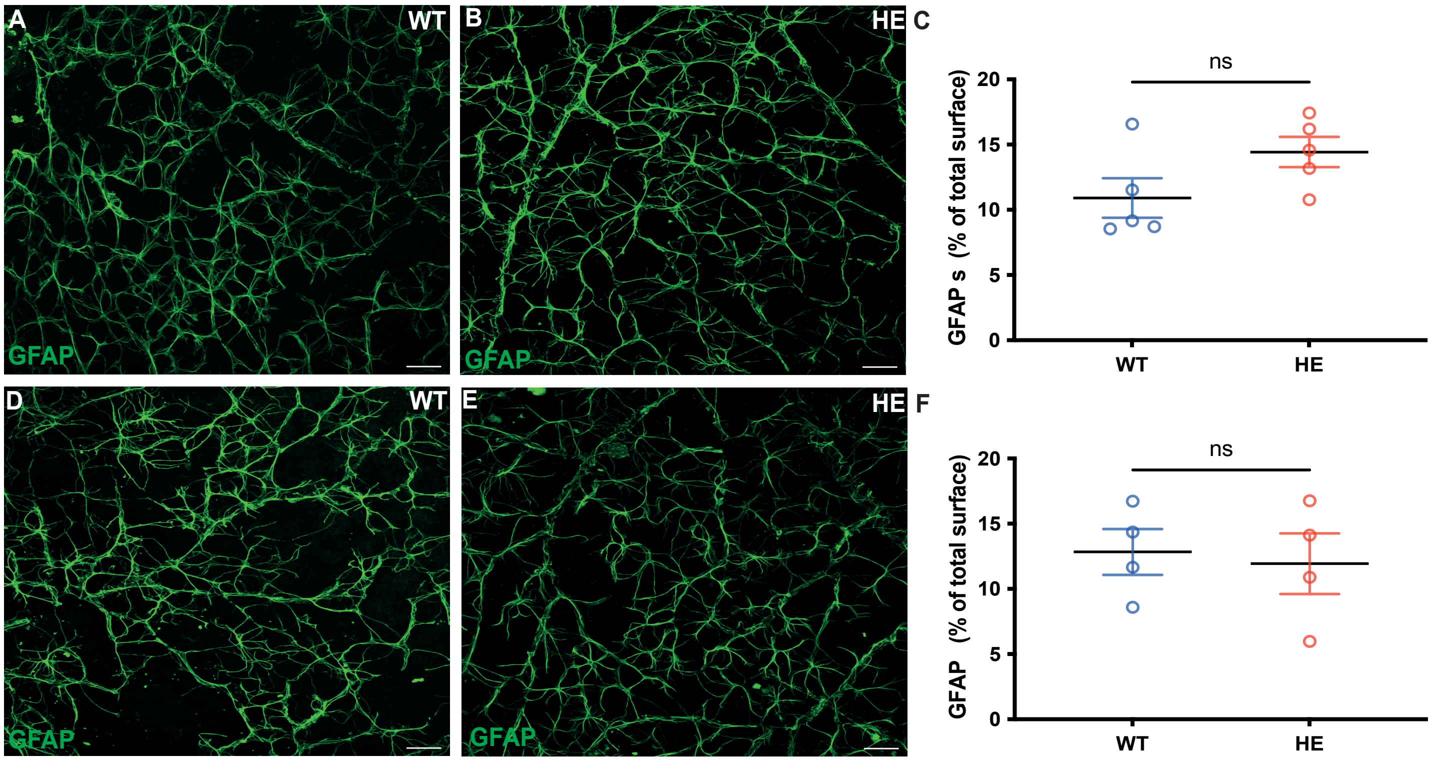

Additional file 6: Fig. S6. Absence of astroglyosis in the retina of P301S mice.
(**A**) Surface coverage by GFAP-immunopositive astrocytes in flat-mount retinas from WT (**A**, **D**) and HE-P301S (**B**, **E**) mice at six- (**A**, **B**) and nine- (**D**, **E**) months of age. Quantification of the surface occupied by GFAP-immunopositive cells at six (**C**) and nine (**F**) months of age. Scale bar=50μm.
