# Supplementary material for: Dysfunction of the glutamatergic photoreceptor synapse in the P301S mouse model of tauopathy
Source: Acta Neuropathol Commun. 2023 Jan 11;11:5. doi: 10.1186/s40478-022-01489-3 (PMC9832799; doi:10.1186/s40478-022-01489-3)
Supplement: Supplementary file 7 — Additional file 7: Table S1. List of antibodies used in the immunofluorescence study. [file 40478_2022_1489_MOESM7_ESM.pdf]

Antibody	Reference	Dilution	Supplier
against phosphorylated Ser202 (AT8)	MN1020	1 :200	Thermo Fisher Scientific
against double-phosphorylated residues Ser214 and Thr212 (AT100)	MN1060	1 :400	Thermo Fisher Scientific
against pathological conformational of tau, in amino acids 7–9 and amino acids 313–322 (MC1)		1 :500	Purchased from Dr. Peter Davies, Albert Einstein University, NY
against tau protein phosphorylated at Ser residues 396/404 (PHF1)		1 :7000	Purchased from Dr. Peter Davies, Albert Einstein University, NY
against RNA-binding protein with multiple splicing (RPBMS)	ABN 1362	1 :500	Merck
against cone-arrestin	AB15282	1 :1000	Merck
against ionized calcium-binding adaptor molecule-1 (Iba1)	W1W019-19741	1 :500	Sobioda
against ionized calcium-binding adaptor molecule-1 (Iba1)	ab5076	1 :500	Abcam
against glial acidic fibrillary protein (GFAP)	LS-B4775-50	1 :500	LSBio
against glutamine synthetase (GlutSyn)	MAB302	1 :300	Merck
against vesicular glutamate transporter-1 (VGlut1)	AB5905	1 :250	Chemicon-Millipore
against vesicular GABA and glycine amino-acid transporter (VIAAT)	PA527569	1 :200	Thermo Fisher Scientific

Additional file 7: Table S1. List of antibodies used in the immunofluorescence study
