# Supplementary material for: Dysfunction of the glutamatergic photoreceptor synapse in the P301S mouse model of tauopathy
Source: Acta Neuropathol Commun. 2023 Jan 11;11:5. doi: 10.1186/s40478-022-01489-3 (PMC9832799; doi:10.1186/s40478-022-01489-3)
Supplement: Supplementary file 1 — Additional file 1: Fig. S1. Distribution of phosphorylated Tau in the retina. (A) Flat-mount retina from a six-month-old HE-P301S mouse, immunolabeled with AT8. (1A) Magnification of region 1 in (A), which is nearest to the optic nerve, followed by 2A and then 3A. (1B). Magnification of region 1, which is nearest to the optic nerve, followed by 2B and 3B of region (B), which shows immunolabeling with PHF1 (C) or MC1 (D) antibodies for all ages. (E) Magnification of a flat-mount retina from a nine-month-old WT mouse, immunolabeled with AT8. Scale bar=100 um. [file 40478_2022_1489_MOESM1_ESM.pdf]

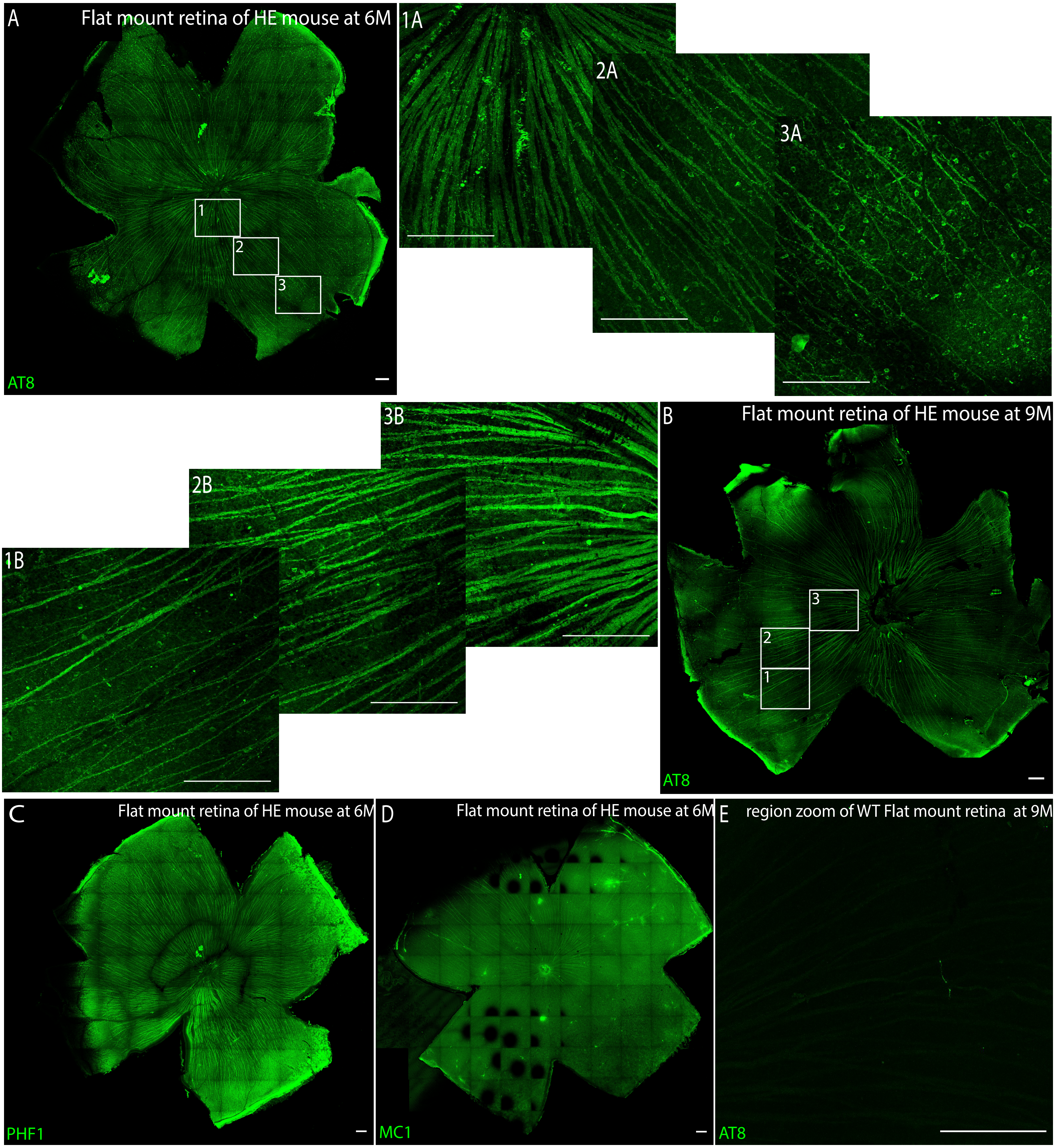

Additional file 1: Fig. S1. Distribution of phosphorylated Tau in the retina. (A): Flat-mount retina from a six-month-old HE-P301S mouse, immunolabeled with AT8. (1A): Magnification of region 1 in (A), which is nearest to the optic nerve, followed by 2A and then 3A. (1B). Magnification of region 1, which is nearest to the optic nerve, followed by 2B and 3B of region (B), which shows immunolabeling with PHF1 (C) or MC1 (D) antibodies for all ages. (E) Magnification of a flat-mount retina from a nine-month-old WT mouse, immunolabeled with AT8. Scale bar=100 um.
